# A Retrospective Investigation on Canine Papillomavirus 1 (CPV1) in Oral Oncogenesis Reveals Dogs Are Not a Suitable Animal Model for High-Risk HPV-Induced Oral Cancer

**DOI:** 10.1371/journal.pone.0112833

**Published:** 2014-11-17

**Authors:** Ilaria Porcellato, Chiara Brachelente, Gabriella Guelfi, Alice Reginato, Monica Sforna, Laura Bongiovanni, Luca Mechelli

**Affiliations:** 1 Department of Veterinary Medicine, University of Perugia, Perugia, Italy; 2 Faculty of Veterinary Medicine, University of Teramo, Teramo, Italy; Georgetown University, United States of America

## Abstract

CPV1 (also called COPV) is a papillomavirus responsible for oral papillomatosis in young dogs. The involvement of this viral type in oral oncogenesis has been hypothesized in oral squamous cell carcinomas (SCCs), but has never been investigated in other neoplastic and hyperplastic oral lesions of dogs. Aim of this study was to investigate the presence of CPV1 in different neoplastic and hyperplastic lesions in order to assess its role in canine oral oncogenesis; according to the results obtained, a second aim of the study was to define if the dog can be considered a valid animal model for oral high risk HPV-induced tumors. Eighty-eight formalin-fixed, paraffin-embedded (FFPE) canine oral lesions including 78 oral tumors (papillomas, SCCs, melanomas, ameloblastomas, oral adenocarcinomas) and 10 hyperplastic lesions (gingival hyperplasia) were investigated with immunohistochemistry for the presence of papillomavirus L1 protein and with Real-Time PCR for CPV1 DNA. RT-PCR for RNA was performed on selected samples. All viral papillomas tested were positive for immunohistochemistry and Real-time PCR. In 3/33 (10%) SCCs, viral DNA was demonstrated but no viral RNA could be found. No positivity was observed both with immunohistochemistry and Real-Time PCR in the other hyperplastic and neoplastic lesions of the oral cavity of dogs. Even though the finding of CPV1 DNA in few SCCs in face of a negative immunohistochemistry could support the hypothesis of an abortive infection in the development of these lesions, the absence of viral RNA points out that CPV1 more likely represents an innocent bystander in SCC oncogenesis. The study demonstrates a strong association between CPV1 and oral viral papillomas whereas viral contribution to the pathogenesis of other oral lesions seems unlikely. Moreover, it suggests that a canine model of CPV1 infection for HPV-induced oncogenesis could be inappropriate.

## Introduction

Papillomaviruses (PVs) are a group of viruses belonging to the *Papillomaviridae* family that are highly species-specific that can affect mammals, birds and reptiles [1]. PVs have a circular double-stranded DNA genome approximately 8 kb in size that typically contains eight ORFs (Open Reading Frames), coding early (E1, E2, E4, E5, E6 and E7) and late (L1 and L2) viral proteins [2], [3]. E1 is a DNA helicase essential for the replication and the amplification of the viral episome in the nucleus of infected cells [4]. E2 proteins participate in the initiation of viral DNA replication, but are pivotal in transcriptional regulation and partitioning of viral genome as well [5]. E6 and E7 oncoproteins are responsible for cellular proliferation and transformation processes, with E7 being recognized as one of the most efficient cell cycle deregulator [6]. E4, together with E5, is involved in the regulation of viral genome amplification and represents the most abundant transcript in productive lesions [7], [8]. Late proteins (L1, L2) encode for the structural proteins of the viral capsid, which are fundamental for the completion of PV life cycle [8]. HPVs (Human Papillomaviruses) are classified into cutaneous and mucosal types based on their tropism for skin or mucosal epithelia, respectively, and can either persist asymptomatically or cause benign hyperproliferative diseases or malignancies [2], [9]. Mucosal HPVs can be subclassified into high-risk and low-risk types with respect to their association to cancer development [10]. This classification was initially based only on epidemiological data and later confirmed with additional evidences from in vitro assays [11]. More than 170 types of HPV have been identified so far, whereas in canine medicine only 14 types of CPVs (Canine Papillomaviruses) are known at this time [12], [13]. The role of CPVs in the pathogenesis of cancer has not been clearly elucidated and none of the canine viral types has been associated to a particular cancer entity. On the contrary, in human medicine the oncogenic role of high-risk HPV types such as HPV 16 and HPV 18 is well known and are regarded to contribute to over 70% of cervical cancers [14]. More recently, HPV infection has been associated also with head and neck cancer, in particular with squamous cell carcinoma of the oropharynx [15], [16]. In dogs, the first PV type documented is the etiological agent of canine oral papillomatosis, originally called COPV (Canine Oral Papillomavirus) and recently re-named CPV1 [2], [17]. The DNA of this virus is 8607 base pairs in length and therefore is the largest PV sequenced [18]. Although the arrangement of CPV1 genome is similar to the other PVs, it lacks the E5 region and it is characterized by a long second non-coding region [19]. Canine oral papillomatosis caused by CPV1 is more frequent in dogs of approximately one year of age and typically causes cauliflower-like exophytic warts; on occasion, tumors can also be fringed or nodular. Lesions are normally localized on the oral mucosa, lips and mucocutaneous junctions, but are not restricted to those sites [17]. They usually undergo spontaneous regression that is associated with a predominant infiltration of CD4+ and CD8+ lymphocytes [20]. Although CPV1 is typically related to benign and spontaneously regressing papillomas, this viral type has been occasionally reported in non-regressing lesions, particularly in oral and perioral squamous cell carcinomas [21], [22]. In the past few years, the presence of HPV DNA has been widely investigated and demonstrated in different oral tumors such as squamous cell carcinoma [23], ameloblastoma [24], salivary gland carcinoma [25], [26], as well as in cutaneous melanoma [27] and other tumors [28], [29], [30]. The aim of this study was to investigate different canine oral tumors and hyperplastic lesions for the presence of CPV1 DNA and RNA as well as viral antigen localization in lesions. Assessing viral prevalence in oral neoplastic events in dogs could provide important insights on the potential role of this specific viral type in canine oral oncogenesis and on the usefulness of the canine model for oral HPV-induced oncogenesis in human beings. This investigation was performed on tumors of epithelial origin, such as squamous cell carcinomas, ameloblastomas and oral adenocarcinomas. A group of melanomas, which are the most common and invasive oral cancers in dogs, was also considered in this study. Moreover, since it is generally accepted that PVs depend on epithelial differentiation for completion of their life cycle [8], this study also included a group of gingival hyperplastic lesions. Routine histology, immunohistochemistry and Real-Time PCR were used in this study. Our study focused on the role of CPV1 in oral oncogenesis through the retrospective immunohistochemical and molecular analysis of a wide range of neoplastic and non-neoplastic oral lesions.

## Materials and Methods

### Ethics statement

This retrospective study was performed on FFPE specimens previously sent to our Departments for histopathologic diagnosis and therefore no approval from ethics committee was needed.

### Sample selection and histopathology

Twenty-two papillomas (Ps), 33 squamous cell carcinomas (SCCs), 11 melanomas (Ms), 10 ameloblastomas (AMs), 2 oral adenocarcinomas (ACs) and 10 cases of gingival hypertrophy/hyperplasia (GH), collected from 2005 to 2012, were selected from the archives of the Department of Veterinary Medicine (University of Perugia, Italy), and of the Faculty of Veterinary Medicine (University of Teramo, Italy). The diagnosis was revised by 2 pathologists on hematoxylin-eosin slides. Papillomas were subsequently classified into viral papillomas (VPs) and squamous papillomas (SPs). VP were diagnosed based on the presence of typical histological features indicative of viral cytopathic effects such as epithelial proliferation, koilocytosis, hypergranulosis, hyperkeratosis and the presence of inclusion bodies.

### Immunohistochemistry

Sections of 2 µm were cut from the 88 formalin-fixed and paraffin-embedded (FFPE) samples and mounted on positive-charged slides. Immunohistochemistry was performed using a rabbit polyclonal antibody against highly conserved L1 capsid protein of BPV1 (1∶200 dilution; Anti-Bovine Papillomavirus/BPV1, BO580 DakoCytomation, Glostrup, Denmark), as reported in other studies [31]. Briefly, FFPE tissue sections were deparaffinized, rehydrated and washed in distilled water. No antigen retrieval was performed. Endogenous peroxidase was blocked with 3% H_2_O_2_ for 8 minutes at room temperature. Slides were then washed in TBS and incubated with the primary antibody for 1 hour in a humidified chamber at room temperature. Slides were incubated with secondary biotinylated universal secondary antibody, followed by sequential incubation with peroxidase-labelled streptavidin (LSAB+/System-HRP, Dako, Glostrup, Denmark). Three-amino-nine-ethylcarbazole was used as chromogen (AEC + Substrate-Chromogen Ready-to-use, Dako, Glostrup, Denmark) and Carazzi’s hematoxylin as counterstain. Coverslips were mounted with aqueous mounting medium. Negative controls were treated in the same manner, omitting the primary antibody and incubating tissue sections with TBS. Positive controls were obtained from bovine cutaneous papillomas.

### Primers design

Probes were designed on the reported sequence of CPV1 [GenBank:L22695.1] in particular on four target ORFs: E4, E7, L1 and L2. Among early viral products, E4 was selected as a target gene because of its high expression (as high as 30% of the total protein content in some productive warts) in productive lesions and E7 because of its recognized oncogenic role [6], [7]. L1 and L2, the late proteins, were included because of their pivotal role in the completion of PV viral cycle [8]. For the detection of each viral gene, forward and reverse primers and probes were designed (Table 1). Probes for E4, E7 and L1 were suitable also for viral RNA detection. The designed primers were verified by NCBI blast analysis and ClustalW alignment analysis to ensure the specificity.

**Table 1:** Primers and probes designed on CPV1 sequence.

Gene	Primer	Probe	Amplicon
**E4**	5′GGAGGAAGCGGAGAATTACCC3′GCTGCTGGTGGTTCGTAGTT	AGCCGCAGCCGTCCTCGTCGT	105 bp
**E7**	5′CGCAACCCTTTTGGATATTGTG3′AGACGATGGTAATTGTTCATAGCA	GACAGAGCAGCCGGAGCCGATAG	79 bp
**L1**	5′GGTTTGGCTTCCTGCACAGA3′CCCACAGTAAGAAGACGTTCACT	ACCTTCCACCACAGCCCAGCACCA	132 bp
**L2**	5′ ATCCTCAAATATGGTAGTGCTGG3′ TTGTGACCCTTGTGCCTAC	ATCAACAGGCAAAGGGGTAGGGG	135 bp
**18S**	5′AAAATTAGAGTGTTCAAAGCAGGC 3′CCTCAGTTCCGAAAACCAACAA	CGAGCCGCCTGGATACCGCAGC	101 bp

### Nucleic acid extraction

DNA and RNA extraction was performed from 5 µm paraffin sections with a commercial kit following the manufacturer’s instructions (Wizard Genomic DNA purification Kit, Promega, Milano, Italia and RecoverAll Total Nucleic Acid Isolation Kit, Ambion, Austin, Texas). Total DNA and RNA were quantified with a fluorometer (Qubit 2.0, Invitrogen, Carlsbad, California) using a high-sensibility commercial kit (Qubit dsDNA HS or RNA HS Assay Kit, Invitrogen, Carlsbad, California) as per the manufacturer’s instructions.

### Reverse Transcription

About 20 ng of total RNA were reverse transcribed in 20 µL of iSCRIPT cDNA (BioRad, Hercules, CA) using random hexamers according to the manufacturer’s suggestions. No-RT controls were included to check for genomic DNA contamination.

### DNA and cDNA Real-Time PCR

According to the manufacturer’s suggestions, 4 µl of DNA (30–50 ng) or 4 µl of cDNA (1–10 ng) were amplified using 12.5 µL of Hot-Rescue Real-Time master mix (Diatheva, Fano, Italy), 0.125 µL of Hot-Rescue DNA Polymerase (Diatheva Fano, Italy), 5 pmoles of probe, 10 pmoles of primers (Integrated DNA Technologies, Coralville, IA), and DNAse/RNAse-free water to 25 µL. All PCR reactions were performed on a Bio-Rad iCycler Real-Time PCR with an initial incubation at 95°C for 15 min, followed by 45 cycles at 95°C for 15 sec and 60°C for 1 min, during which fluorescence data were collected. Each sample was run in triplicate and the results were averaged. The Ct was automatically computed by each curve. Sample amplification fidelity was verified by agarose gel electrophoresis. PCR products were purified and sequenced by QIAquick PCR Purification Kit (Quiagen, Valencia, CA). The 18S was used as endogenous control to normalize sample variations in the amount of starting RNA samples as previously reported [32]. For each PCR run, no template controls and noRT controls were included in order to ascertain the absence of contaminating gDNA.

## Results

### Histology and immunohistochemistry

**Figure 1:**
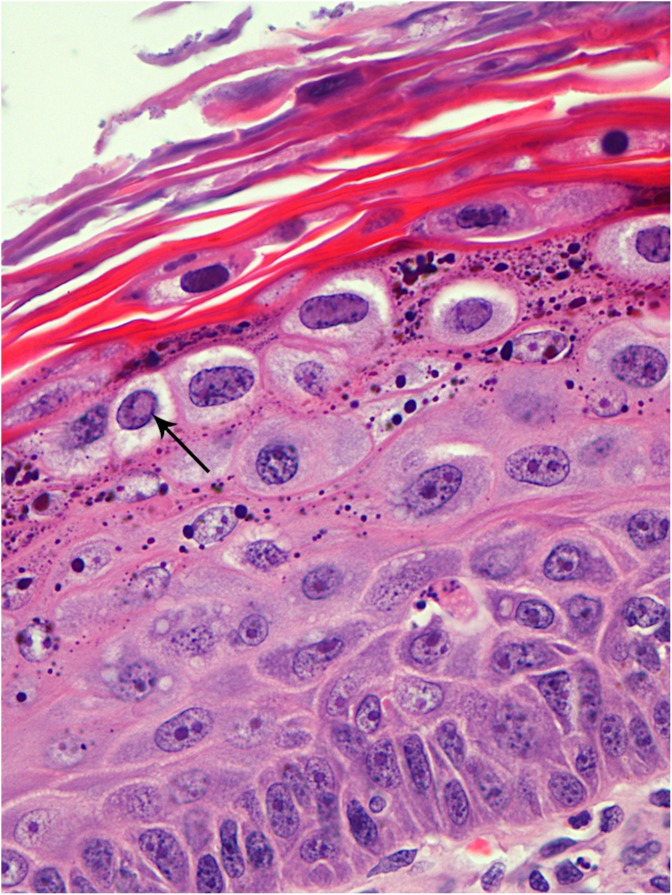
Histological characteristics of canine oral viral papilloma: hyperkeratosis, hypergranulosis, koilocytes and inclusion bodies (arrow). H&E; 40x.

**Figure 2:**
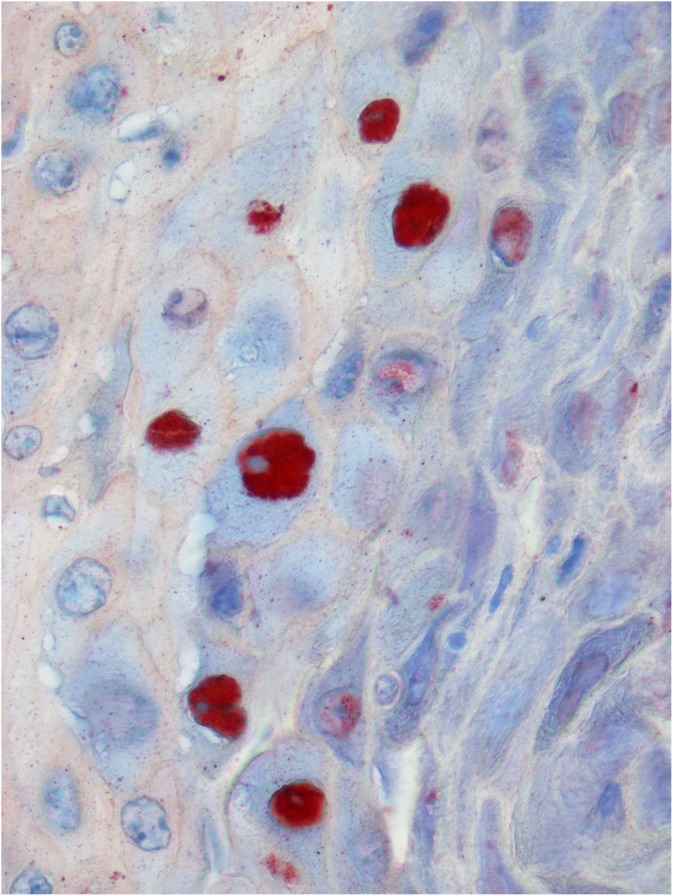
Immunohistochemical staining with intranuclear positivity for L1 protein. AEC and Carazzi’s hematoxylin; 40x.

With histological examination on routinely stained sections, papillomas were classified into VPs (15/22) and SPs (7/22) evaluating the presence of characteristic cytopathic features of PV infection (epidermal proliferation, koilocytosis, hypergranulosis, hyperkeratosis and inclusion bodies) (Figure 1). One case with mild hypergranulosis and focal hyperkeratosis was difficult to classify due to the lack of inclusion bodies and of koilocytes. However, after serial sectioning, occasional koilocytes were observed and the case was classified as a VP. Histological characteristics of SPs and VPs are shown in Table S1. Positive intranuclear immunostaining confirmed all VPs previously diagnosed on HE stained sections (Figure 2). Positive cells were localized especially in the interdigitating epidermis and often in areas where the epithelium was characterized by severe hypergranulosis. Only in one case, the immunolabelling was predominantly seen in superficial desquamating cells. All the other tumors and hyperplastic lesions were negative for PV immunostaining. Nevertheless, a finely granular cytoplasmic staining was detected in five cases of SCCs.

### Real-Time PCR amplification of DNA and RNA

DNA extraction yield ranged from 50 to 90 ng/µl and RNA concentration was about 20 ng/µl. Confirming the immunohistochemical results, Real-Time PCR detected viral DNA in all 15 VPs. VPs were positive for all the four probes specifically designed for the detection of CPV1. Three SCCs out of 33 resulted positive for the presence of CPV1 DNA with all the four probes tested. No overlapping with the five SCCs with finely cytoplasmic immunostaining was evidenced. Sequencing of all amplicons demonstrated a complete identity (100%) to the sequence in GenBank database (accession number L22695.1). No viral DNA was amplified in all AMs, Ms, ACs and GH tested. Real-Time PCR for cDNA was performed on a selection of 5 VPs, 5 SPs, 8 SCCs, 3 of which were the ones positive for CPV1 viral DNA. The criteria of selection of the samples to be tested was based on the availability of material for RNA extraction, after previous tests had been performed. For the 3 cases of SCCs positive for viral DNA, the quantity of tissue was limited (<1 mm^2^ sections). In these cases, total mRNA quantity was extremely low (about 1 ng/µl) due to scarce residual tissue after previous tests. Viral papillomas were used as a positive control and viral mRNA was retrieved in all of them. The three SCCs positive for viral DNA were instead negative. Extraction of RNA and assay experiments were repeated twice in order to assess the result and rule out environmental contamination. Results of Real-Time PCR are shown in Table 2.

**Table 2:** DNA and mRNA levels of CPV1 measured by Real-Time PCR.

DNA	Cases	E4	E7	L1	L2
VPs	15/15	5.01±0.85	3.00±0.74	4.75±0.99	6.50±0.90
SPs	7/7	ND	ND	ND	ND
SCCs	3/33	6.23±0.65	7.07±0.73	7.38±0.81	7.53±0.44
	30/33	ND	ND	ND	ND
Ms	11/11	ND	ND	ND	ND
AMs	10/10	ND	ND	ND	ND
ACs	2/2	ND	ND	ND	ND
GH	10/10	ND	ND	ND	ND
NC	5/5	ND	ND	ND	ND
PC	5/5	3.20±1.14	2.04±0.70	2.95±0.70	3.80±0.84
**RNA**	**CASES**	**E4**	**E7**	**L1**	
VPs	5/5	12.8±0.84	11.8±0.84	13.8±1.30	
SPs	5/5	ND	ND	ND	
SCCs (CPV1+)	3/3	ND	ND	ND	
SCCs (CPV1−)	5/5	ND	ND	ND	
NC	5/5	ND	ND	ND	
PC	5/5	6.80±1.95	5.8±1.09	7.80±1.30	

mRNA was extracted only from those samples whose DNA was tested (5 out of 15 for VPs; 5 out of 15 for SPs and 8 out of 33 for SCCs). E4, E7, L1 and L2 probes were used to detect CPV1 DNA and E4, E7 and L1 probes were suitable for viral mRNA detection. Ct values were normalized with housekeeping 18S. In the table Ct values are shown as the means ± standard deviation. Not detectable Ct are indicated as ND. The positive (PC) and negative controls (NC) ensured the specificity of the results. No Template Controls were included on every RNA plate for every probe to check for contamination.

### Histological characteristics of CPV1-associated SCCs

The examination of CPV1 positive SCCs did not show peculiar histological characteristics that could be suspicious of papillomavirus infection, compared to the group of CPV1 negative SCCs (Table S2).

## Discussion

The present study evaluated the presence of CPV1 in different hyperplastic/neoplastic canine oral lesions, in order to investigate the role of this virus in oral oncogenesis. In human medicine, the oncogenetic role of high-risk HPVs has been demonstrated for cervical cancer [16] and more awareness is growing for their role in the pathogenesis of head and neck SCCs [15]. The increasing consideration paid to the study of PVs in veterinary medicine is justified both by the growing attention for animal health and by the possibility of using the dog as an experimental model to study the interaction between PV infection and carcinogenesis in humans [33], [34]. In spite of this, little information is available about the prevalence of CPVs in canine tumors. With this study we pointed out that CPV1 is strongly associated with VPs in dogs in oral and perioral sites. Moreover, we observed the presence of viral DNA only in three SCCs, whereas no viral mRNA was found to demonstrate viral activity. This result confirms what is described in the veterinary literature [22], [35] about the low potential of malignant transformation associated with CPV1 infection. Based on our data, the dog should not be used as a model for the study of malignant lesions caused by high-risk genotypes of HPVs.

Results obtained from the histological evaluation of all the oral papillomas included in the present study showed that koilocytosis and hypergranulosis were more evident in VPs compared to SPs. However, 2/7 SPs showed mild or moderate hypergranulosis, so this morphological aspect alone cannot be considered a reliable indicator of viral infection. According to our results, the presence of koilocytes and inclusion bodies represents the most important histopathologic sign of the viral presence. Immunohistochemistry was useful not only to confirm the histological classification and to evaluate the stage of infection, but was particularly helpful in cases of late phase PV infection where koilocytosis and inclusion bodies were not evident histologically. In these cases, immunolabeling was confined to the desquamating cells of the epidermis. The cytoplasmic positivity encountered in five SCCs, particularly in areas of squamous differentiation, was interpreted as an artifact, due to the fact that these tumors were negative for DNA amplification.

Real-Time PCR confirmed the presence of both CPV1 DNA in all the 15 VPs of the study and of viral mRNA in the five selected VPs tested. This confirms a strong correlation between CPV1 and VPs. In our study, most VPs were detected on mucocutaneous junctions and only one case was biopsied from the skin of the chin, in agreement with previous studies reporting the possible presence CPV1 in cutaneous lesions [17], [22], [36]. Therefore CPV1 seems to show a less marked tropism for mucous membranes compared to the Alpha-HPVs, which comprise high-risk types. Alpha-HPVs infections indeed are generally restricted to oral mucosa and can be rarely found in cutaneous lesions [37], [38], [39].

Despite the absence of PV-immunolabelling, PCR revealed the presence of viral DNA in 3/33 SCCs tested. This limited number of CPV1 DNA positive cases does not allow us to draw statistically significant conclusions about possible histomorphologic criteria that can be associated with the presence of the virus. Each of the three cases showed different histological degrees of differentiation, cellular atypia and inflammation. Worthy of note is that the search for viral mRNA in these three SCCs positive for CPV1 DNA did not yield positive results. Different hypotheses can be made to justify this result. First of all, the amount of material from FFPE biopsies of SCCs was extremely limited when cut for RNA extraction and this may have resulted in an excessively low amount of viral mRNA on total mRNA extracted, not allowing amplification by Real-Time PCR. On the other hand, since positivity for viral mRNA was found in all VPs used as a positive control, we can assume that CPV1 could be interpreted as an innocent bystander in the context of SCCs. Even though healthy skin of humans and dogs can function as a potential reservoir for PV infection [40], [41] specimens of this study had been previously treated with formalin, therefore the hypothesis of a superficial contamination seems unlikely.

A negative PV-immunolabelling associated with negative Real-Time PCR results (both for viral DNA and mRNA) in all the other lesions (hyperplastic and neoplastic) included in the present study suggests that CPV1 has likely no role in the pathogenesis of canine oral lesions different from VPs.

In conclusion, with this study we investigated the role of CPV1 in a wide range of different neoplastic and hyperplastic lesions arising in the oral cavity in the dog. CPV1 was strongly associated with VPs. Since the presence and the transcriptional activity of CPV1 have only been proven for VPs, it seems unlikely that CPV poses a high risk for neoplastic progression. In addition to expanding the number of cases tested for the presence of CPV1, it would be important to investigate the role of other types of CPVs in the pathogenesis of the most common cancers of the oral cavity in dogs. Considering our results, we believe that CPV1-induced lesions have a low risk of malignant progression and that the use of CPV1 as a model for HPV-associated head and neck cancer is consequently not supported by our data, especially as the incidence of CPV1 in the tested SCCs was very low.

## Supporting Information

Table S1 **Histological characteristics of the 22 squamous and viral papillomas in this study.** Features of different tumors were graded: 1 = weak/mild; 2 = moderate; 3 = high/severe.
(DOCX)

Table S2 **Histological characteristics of the 33 SCCs of this study.** IB = inclusion bodies; IHC = immunohistochemistry. Features of different tumors were graded: 1 = weak/mild; 2 = moderate; 3 = high/severe. Immunohistochemistry assays with an uncertain result (?) were classified as negative and uncertain positivity encountered was considered as an artifact. N/A = not performed due to scant material available for RNA extraction.
(DOCX)
