# Correction: A Retrospective Investigation on Canine Papillomavirus 1 (CPV1) in Oral Oncogenesis Reveals Dogs Are Not a Suitable Animal Model for High-Risk HPV-Induced Oral Cancer

**DOI:** 10.1371/journal.pone.0118051

**Published:** 2015-02-11

**Authors:** The PLOS ONE Staff

In the Author Contributions section, Gabriella Guelfi (GG) should be listed as one of the persons who analyzed the data, and as one of the persons who contributed to the writing of the manuscript.
