# Supplementary material for: A Retrospective Investigation on Canine Papillomavirus 1 (CPV1) in Oral Oncogenesis Reveals Dogs Are Not a Suitable Animal Model for High-Risk HPV-Induced Oral Cancer
Source: PLoS One. 2014 Nov 17;9(11):e112833. doi: 10.1371/journal.pone.0112833 (PMC4234530; doi:10.1371/journal.pone.0112833)
Supplement: Table S1 — Histological characteristics of the 22 squamous and viral papillomas in this study. Features of different tumors were graded: 1 = weak/mild; 2 = moderate; 3 = high/severe. (DOCX) [file pone.0112833.s001.docx]

| N. | Classification | Hyperkeratosis | Inflammation | Hypergranulosis | Koilocytosis | Inclusion bodies |  |  |
| --- | --- | --- | --- | --- | --- | --- | --- | --- |
| 1 | SP | 3 | 0 | 1 | 0 | N |  |  |
| 2 | SP | 1 | 3 | 0 | 0 | N |  |  |
| 3 | SP | 1 | 2 | 0 | 0 | N |  |  |
| 4 | SP | 0 | 2 | 0 | 0 | N |  |  |
| 5 | SP | 0 | 2 | 0 | 0 | N |  |  |
| 6 | SP | 0 | 2 | 0 | 0 | N |  |  |
| 7 | SP | 1 | 2 | 2 | 0 | N |  |  |
| 8 | VP | 1 | 1 | 2 | 2 | Y |  |  |
| 9 | VP | 2 | 1 | 3 | 3 | Y |  |  |
| 10 | VP | 2 | 0 | 3 | 3 | Y |  |  |
| 11 | VP | 1 | 1 | 1 | 3 | Y |  |  |
| 12 | VP | 3 | 2 | 1 | 1 | Y |  |  |
| 13 | VP | 1 | 1 | 1 | 1 | N |  |  |
| 14 | VP | 2 | 2 | 2 | 2 | Y |  |  |
| 15 | VP | 2 | 2 | 3 | 2 | Y |  |  |
| 16 | VP | 2 | 1 | 2 | 1 | N |  |  |
| 17 | VP | 3 | 3 | 1 | 1 | Y |  |  |
| 18 | VP | 3 | 2 | 3 | 2 | Y |  |  |
| 19 | VP | 3 | 3 | 3 | 1 | N |  |  |
| 20 | VP | 2 | 3 | 2 | 3 | Y |  |  |
| 21 | VP | 3 | 1 | 2 | 2 | Y |  |  |
| 22 | VP | 2 | 0 | 2 | 1 | N |  |  |
|  |  |  |  |  |  |  |  |  |
