# Supplementary material for: A Retrospective Investigation on Canine Papillomavirus 1 (CPV1) in Oral Oncogenesis Reveals Dogs Are Not a Suitable Animal Model for High-Risk HPV-Induced Oral Cancer
Source: PLoS One. 2014 Nov 17;9(11):e112833. doi: 10.1371/journal.pone.0112833 (PMC4234530; doi:10.1371/journal.pone.0112833)
Supplement: Table S2 — Histological characteristics of the 33 SCCs of this study. IB = inclusion bodies; IHC = immunohistochemistry. Features of different tumors were graded: 1 = weak/mild; 2 = moderate; 3 = high/severe. Immunohistochemistry assays with an uncertain result (?) were classified as negative and uncertain positivity encountered was considered as an artifact. N/A = not performed due to scant material available for RNA extraction. (DOCX) [file pone.0112833.s002.docx]

| N. | Cellular differentiation | Cellular atypia | Mitoses/10 HPF | Horn pearls | Inflammation | Hyperkeratosis | Koilocytosis | Hypergranulosis | IB | IHC | Real-Time PCR  for CPV1 DNA | Real-Time PCR  for CPV1 RNA |
| --- | --- | --- | --- | --- | --- | --- | --- | --- | --- | --- | --- | --- |
| 1 | 3 | 1 | 1 | 0 | 1 | 0 | 0 | 0 | N | - | - | N/A |
| 2 | 2 | 2 | 3 | 2 | 2 | 1 | 1 | 0 | N | ? | - | N/A |
| 3 | 1 | 2 | 8 | 0 | 1 | 0 | 0 | 0 | N | - | - | N/A |
| 4 | 3 | 1 | 8 | 0 | 3 | 1 | 0 | 0 | N | - | - | N/A |
| 5 | 1 | 3 | 19 | 0 | 2 | 0 | 1 | 0 | N | - | - | N/A |
| 6 | 3 | 1 | 5 | 3 | 2 | 0 | 0 | 1 | N | ? | - | - |
| 7 | 2 | 2 | 9 | 1 | 2 | 0 | 0 | 0 | N | - | - | N/A |
| 8 | 3 | 1 | 4 | 1 | 2 | 0 | 0 | 1 | N | - | - | - |
| 9 | 2 | 2 | 15 | 0 | 1 | 1 | 0 | 0 | N | - | - | - |
| 10 | 1 | 3 | 45 | 0 | 2 | 1 | 0 | 0 | N | - | - | N/A |
| 11 | 3 | 2 | 50 | 2 | 2 | 0 | 0 | 0 | N | - | - | N/A |
| 12 | 2 | 1 | 6 | 0 | 1 | 0 | 0 | 0 | N | - | - | - |
| 13 | 3 | 1 | 7 | 3 | 2 | 1 | 0 | 0 | N | ? | - | N/A |
| 14 | 3 | 1 | 9 | 2 | 3 | 0 | 0 | 2 | N | ? | - | N/A |
| 15 | 2 | 2 | 8 | 1 | 0 | 0 | 1 | 1 | N | - | - | N/A |
| 16 | 2 | 1 | 2 | 0 | 2 | 0 | 0 | 0 | N | - | - | N/A |
| 17 | 2 | 3 | 8 | 1 | 3 | 0 | 0 | 0 | N | - | - | N/A |
| 18 | 3 | 1 | 8 | 1 | 3 | 0 | 1 | 0 | N | - | - | N/A |
| 19 | 2 | 2 | 10 | 0 | 2 | 1 | 2 | 0 | N | - | - | - |
| 20 | 3 | 2 | 3 | 1 | 3 | 0 | 0 | 0 | N | - | - | N/A |
| 21 | 2 | 1 | 4 | 0 | 1 | 1 | 0 | 0 | N | - | + | - |
| 22 | 1 | 3 | 5 | 0 | 0 | 0 | 0 | 0 | N | - | - | N/A |
| 23 | 2 | 2 | 8 | 0 | 3 | 0 | 0 | 0 | N | - | - | N/A |
| 24 | 3 | 2 | 18 | 1 | 2 | 0 | 1 | 0 | N | - | + | - |
| 25 | 2 | 1 | 13 | 1 | 1 | 0 | 0 | 0 | N | - | + | - |
| 26 | 1 | 3 | 12 | 0 | 3 | 1 | 0 | 0 | N | - | - | N/A |
| 27 | 1 | 3 | 14 | 0 | 1 | 0 | 0 | 0 | N | - | - | N/A |
| 28 | 2 | 2 | 10 | 0 | 2 | 2 | 2 | 0 | N | - | - | N/A |
| 29 | 2 | 3 | 16 | 1 | 3 | 0 | 0 | 0 | N | ? | - | N/A |
| 30 | 2 | 3 | 18 | 0 | 3 | 0 | 0 | 0 | N | - | - | N/A |
| 31 | 3 | 1 | 9 | 1 | 3 | 0 | 0 | 0 | N | - | - | N/A |
| 32 | 3 | 1 | 1 | 0 | 1 | 0 | 0 | 0 | N | - | - | N/A |
| 33 | 1 | 2 | 21 | 0 | 3 | 0 | 1 | 0 | N | - | - | N/A |
